# Alterations in Circulating MicroRNAs and the Relation of MicroRNAs to Maximal Oxygen Consumption and Intima–Media Thickness in Ultra-Marathon Runners

**DOI:** 10.3390/ijerph18147234

**Published:** 2021-07-06

**Authors:** Ceren Eyileten, Alex Fitas, Daniel Jakubik, Pamela Czajka, Anna Mróz, Anna Czajkowska, Katarzyna Witek, Wawrzyniec Bakalarski, Salvatore De Rosa, Marek Postuła, Łukasz A. Małek

**Affiliations:** 1 Center for Preclinical Research and Technology CEPT, Department of Experimental and Clinical Pharmacology, Medical University of Warsaw, 02-097 Warsaw, Poland; ceyileten@wum.edu.pl (C.E.); fitas.alex2@qmail.com (A.F.); djakubik@wum.edu.pl (D.J.); czajka.pamela@gmail.com (P.C.); 2 Department of Physical Education, Józef Piłsudski University of Physical Education in Warsaw, 00-809 Warsaw, Poland; anna.mroz@awf.edu.pl; 3 Department of Tourism and Recreation, Józef Piłsudski University of Physical Education in Warsaw, 00-809 Warsaw, Poland; anna.czajkowska@awf.edu.pl (A.C.); katarzyna.witek@awf.edu.pl (K.W.); wawrzyniec.bakalarski@awf.edu.pl (W.B.); 4 Division of Cardiology, Department of Medical and Surgical Sciences, “Magna Graecia” University, 88100 Catanzaro, Italy; saderosa@unicz.it; 5 Department of Epidemiology, Cardiovascular Disease Prevention and Health Promotion, Institute of Cardiology, 04-628 Warsaw, Poland; lmalek@ikard.pl

**Keywords:** microRNA, miRNA, endurance sport, cardiovascular disease, atherosclerosis

## Abstract

The impact of long-term training on cardiovascular disease (CVD) is not clear. Carotid intima–media thickness (CIMT) test is recommended as a useful measure to diagnose the early stages of atherosclerosis. MicroRNAs (miRNAs) are altered due to endurance exercise and can be promising biomarkers of pathophysiological changes. We aimed to evaluate the association of circulating miRNAs with physical fitness and markers of atherosclerosis in ultra-marathon runners. Ultra-marathon runners had 28-fold upregulation of miR-125a-5p expressions compared to control individuals (*p* = 0.002), whereas let-7e and miR-126 did not differ statistically between ultra-marathon runners and controls. In the ultra-marathon runners’ group, negative correlations were observed between VO_2_max/kg and relative expression of miR-125a-5p and miR-126 (r = −0.402, *p* = 0.028; r = −0.438, *p* = 0.032, respectively). Positive correlations were observed between CIMT and miR-125a-5p and miR-126 (r = 0.388, *p* = 0.050; r = 0.504, *p* = 0.023, respectively) in ultra-marathon runners. Individuals with the highest quartile of VO_2_max/kg had 23-fold lower miR-126 expression in comparison to subgroups with lower VO_2_max/kg (*p* = 0.017). Our results may indicate that both miRNAs may serve as a biomarker for early pathological changes leading to atherosclerosis burden in athletes. Furthermore, the association between miRNAs and traditional risk factors for CVD indicate a possible use of these molecules as early biomarkers of future cardiovascular health.

## 1. Introduction

Long-term endurance training can be defined as workouts performed 10–12 times a week and lasting for 15–30 h in case of elite athletes, or 4–5 times for 6–10 h in case of recreational athletes for a longer period of time. Additionally, in both groups, 80% of exercise is performed with the intensity below the lactate threshold, which means with 45–80% of maximal oxygen consumption (VO_2_max) for more than 60 min, and 20% of high intensity-interval training (HIIT) (lasting 15–90 min per session) [1,2]. This type of training can induce several physiological adaptations in cardiovascular systems, such as balanced enlargement of heart chambers and mild myocardial hypertrophy [3]. Long-term endurance training is also related to enhanced VO_2_max [4,5,6], which leads to increased cardiac output and therefore improves physical fitness [7] and has a protective effect against cardiovascular disease (CVD) [8]. On the other hand, several recent studies suggested that ultra-endurance training can function as a double-edged sword. For example, HIIT is related to muscle hypoxia which may cause oxidative stress, reactive oxygen forms production and enhance inflammation [2,9]. Additionally, high-volume, high-intensity exercise training may induce atherosclerosis due to increased atherosclerotic lesions, however, the exact mechanism is still unknown [10,11,12,13]. The early diagnosis of atherosclerosis before the onset of clinical signs and symptoms is difficult. Carotid intima–media thickness (CIMT) test results were found related to cardiovascular risk, thus CIMT was recommended as a potentially useful diagnostic tool in early stages of atherosclerosis [14,15,16,17,18].

MicroRNAs (miRNAs) are a class of small non-coding RNAs that function as regulators of post-transcriptional gene expressions. They can influence cell development, proliferation, differentiation and apoptosis. Moreover, miRNAs may have a relevant regulatory potential in atherosclerosis and pathophysiology of CVDs, through regulating cardiomyocyte hypertrophy, fibrosis and injury [11,19,20,21]. Importantly, circulating miRNAs are found altered due to endurance exercise. For example, a previous study found circulating miR-126 increased after different forms of endurance exercise in healthy subjects [22]. Moreover, Nielsen et al. showed that acute exercise induced decreases in circulating miR-221 and miR-146a [23], whereas Baggish et al. documented those miRNAs as increased following acute exhaustive exercise [24]. MiRNAs can be promising biomarkers of the cardiovascular physiological adaptations and possible pathophysiological changes due to their high stability in blood plasma and serum [11,23,25,26,27,28]. However further analysis of miRNAs’ utility as biomarkers is required as several contradictory results were presented. 

MiR-126 deserves special attention as studies show that it can have favorable effects on vascular integrity, angiogenesis, and stabilization of atherosclerotic plaques [22,29,30]. Increased expression of miR-126 endorses a more stabilized plaque phenotype and therefore correlates with a significantly lower frequency of cardiovascular events in patients with coronary artery disease [30,31]. Similarly, some previous studies indicated that miR-125a-5p expressions were decreased in atherosclerotic plaques of coronary atherosclerosis patients [32,33]. However, the underlying molecular mechanism of both miR-126 and miR-125a-5p in the progression of atherosclerosis is still poorly understood. Furthermore, endurance training is associated with increased markers of inflammation following a bout of exercise, and it was suggested that acute endurance exercise causes induction of circulating inflammatory miRNA expression, such as Let-7 family miRNAs [34,35]. 

Briefly, ultra-endurance exercise is characterized by cardiovascular adaptation. Importantly, several clinical studies on ultra-marathon runners reported that intensive training can be also related to cardiovascular pathological changes [36,37,38,39]. However, results are contradictory [40]. Several molecular based studies showed that endurance training may increase oxidative stress, hypoxia and cardiac fibrosis [41,42]. On the other hand, the research of circulating miRNAs involved in response to chronic endurance training is still very limited and study on the influence of extreme high intensity training is missing. Knowledge of the circulating miRNAs levels can advance exercise assessment and may facilitate differential diagnosis concerning adaptive and pathological changes [43]. Consequently, this study aimed to evaluate the miRNAs expression difference between ultra-marathon runners and non-athletic healthy subjects, as well as the association of miR-126, Let-7e and miR-125a-5p with physical fitness (measured with maximal oxygen consumption) and markers of atherosclerosis (CIMT) in ultra-marathon runners. 

## 2. Materials and Methods

### 2.1. Study Group 

The Ethics Committee of the Regional Medical Chamber in Warsaw approved both the study protocol and the informed consent form (no 52/17) [44]. The study was conducted in accordance with the current version of the Declaration of Helsinki at the time when the study was designed, and informed written consent from all participants was obtained. In this study we included 30 healthy, male, ultra-marathon runners and age-matched non-athletic healthy individuals (*N* = 9). The full characterization of the study population was published previously [5,6]. Briefly, in the present analysis we included runners with numerous years of documented training, running at least 70 km a week and often participating in ultra-marathon competitions. The detailed medical history of enrolled participants was taken, together with their body mass index (BMI), baseline electrocardiography (ECG), blood pressure (BP) measurement at rest, carotid ultrasound and whole blood count as well as creatinine concentration measurement on the day, but before cardiovascular magnetic resonance imaging. Subsequently all participants underwent cardiovascular magnetic resonance (CMR) with parametric imaging (with pre-contrast T1—time after magnetization inversion and T2—time constant for loss of transverse magnetization-mapping, Siemens Magnetom Skyra 3 T scanner) and gadolinium contrast administration followed by late gadolinium enhancement (LGE) and post-contrast T1-mapping (Siemens, Erlangen, Germany). A cardiopulmonary exercise test (CPET), by using (Saturn, h/p/cosmos, Nussdorf—Traunstein, Germany and Metamax 3B, Cortex Biophysik GmbH, Leipzig, Germany) was performed within 4 weeks of the CMR according to the protocols as described previously [5,6].

### 2.2. Blood Collection

Approximately 9 mL of blood was obtained from antecubital vein using a plasma separator tube between 12 and 24 h after the last training. Participants were informed to withhold from intensive exercise for 24 h before the blood sampling. Withdrawn blood was kept at room temperature for 30 min. Blood collection tubes were centrifuged at 1500× *g* for 15 min at 18–25 °C. Plasma was aliquoted into 500 μL volumes and stored in −80 °C freezer until the laboratory analysis.

### 2.3. RNA Preparation, Detection, and Quantification of miRNAs by Quantitative PCR

In order to purify samples from cell debris, plasma samples after thawing at room temperature were subjected to centrifugation at 16,000× *g* for 10 min at 4 °C. Total RNA was isolated using miRVana PARIS Kit (invitrogen, Applied technologies, Carlsbad, CA, USA) from 500 μL of plasma and diluted at a ratio of 1:10. Subsequently, the obtained RNA template was subjected to a reverse transcription reaction using the TaqMan miRNA Reverse Transcription kit (Applied Biosystems, Foster City, CA, USA) according to guidelines provided by the manufacturer. Afterwards, 3 μL of the product was used to detect miRNA expression by quantitative polymerase chain reaction (qPCR) using TaqMan miRNA Assay kits (ABI, Vernon, CA, USA) for the corresponding miRNAs on a The CFX384 Touch Real-Time PCR Detection System (BioRad Inc., Hercules, CA, USA). Cel-miR-39 was added as an exogenous spike-in control. Mean values of all reactions performed in triplicate were used in statistical analysis [45,46].

### 2.4. Statistical Analysis

All results for categorical variables were presented as a number and a percentage. Continuous variables were expressed as mean and standard deviation (SD) or median and interquartile range (IQR), depending on the normality of distribution assessed with the use of the Shapiro–Wilk test. Student’s *t*-test or the Mann-Whitney test for unpaired samples were applied to compare cases and controls depending on the normality of the distribution. To assess the correlation between continuous variables, a Spearman test was applied. Receiver operating characteristic (ROC) curves were used to analyze the relation between miRNAs expressions and maximal intima–media thickness. All tests were two-sided with the significance level of *p* < 0.05. Calculations were performed using SPSS version 22.0 (IBM Corporation, Chicago, IL, USA).

## 3. Results

### 3.1. Participants

Thirty male ultra-marathon runners and nine healthy, non-athletic male subjects were enrolled in this study. The whole cohort completed all required tests. We did not observe any significant differences at the baseline demographics regarding age, anthropometric measures and blood pressure between the studied and control group. Detailed demographic data are presented in Table 1 and Table 2 (also previously provided [5,6]).

### 3.2. Circulating miRNAs

**Figure 1:**
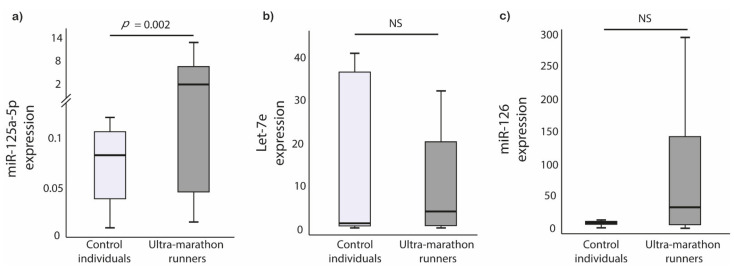
miRNAs relative expression difference between the groups. (**a**) miR-125a-5p; (**b**) Let-7e; (**c**) miR-126.

Circulating miRNAs expression was determined in the blood plasma. Ultra-marathon runners had 28-fold upregulation of miR-125a-5p expressions compared to control individuals (*p* = 0.002), whereas let-7e and miR-126 did not differ statistically between ultra-marathon runners and controls (*p* = 0.806; *p* = 0.140, respectively) (Figure 1). 

### 3.3. Correlations between miRNAs and Clinical Parameters

Correlation analysis was performed by using the Spearman test. In the ultra-marathon runners’ group, negative correlations were observed between VO_2_max/kg and relative expression of circulating miR-125a-5p and miR-126 (r = −0.402, *p* = 0.028; r = −0.438, *p* = 0.032, respectively). Positive correlations were observed between CIMT and circulating miR-125a-5p and miR-126 (r = 0.388, *p* = 0.050; r = 0.504, *p* = 0.023, respectively) in ultra-marathon runners. 

### 3.4. Carotid Intima–Media Thickness and miRNAs Expression

**Figure 2:**
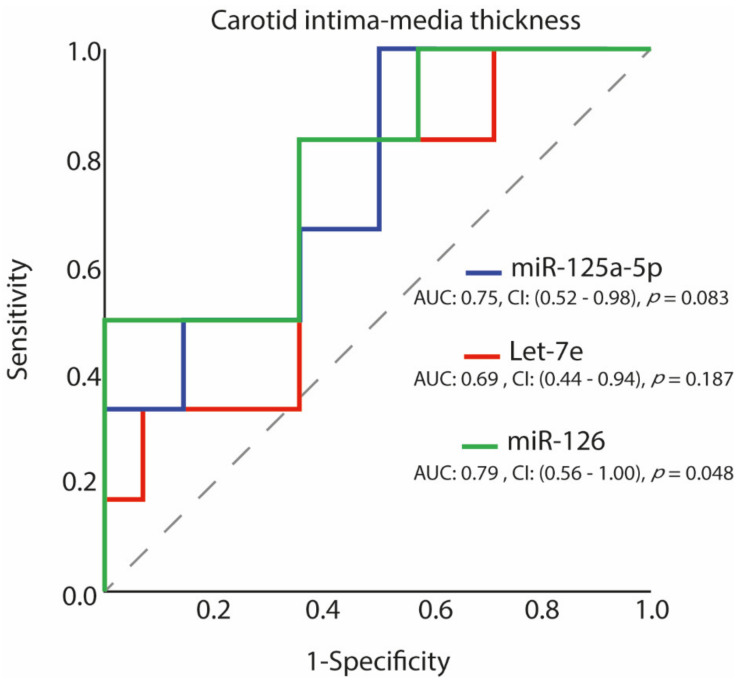
Receiver operating characteristic (ROC) curves of miR-125a-5p, Let-7e, miR-126 prediction of increased CIMT.

**Figure 3:**
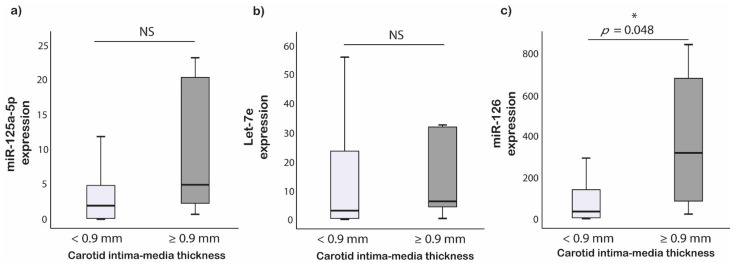
MiRNAs expression difference between CIMT groups in ultra-marathon runners. (**a**) miR-125a-5p; (**b**) Let-7e; (**c**) miR-126, * Comparison is significant at the 0.05 level.

ROC analysis showed that miR-126 levels have a good diagnostic performance to predict subjects with increased CIMT. In fact, the area under the curve (AUC) for miR-126 was 0.8 (Figure 2). miR-126 expression levels greater than or equal to the optimal cut-off of 70.6 identified by means of the maximization of the Youden’s function had sensitivity of 83%, specificity of 64%, a negative predictive value of 90%, and a positive predictive value of 50% for the prediction of increased CIMT. Ultra-marathon runners with CIMT > 0.9 mm had 10-fold increased relative expression of miR-126 compared to those with CIMT < 0.9 mm (*p* = 0.048) (Figure 3).

### 3.5. Cardiopulmonary Fitness and miRNAs Expression

**Figure 4:**
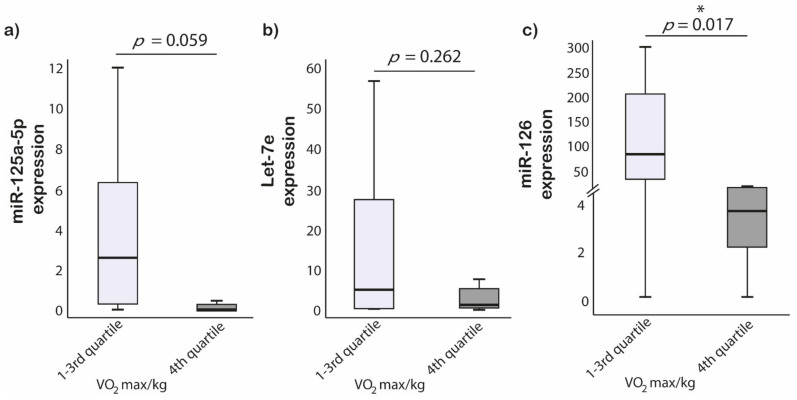
MiRNAs expression difference between fourth quartile and lower quartiles of VO_2_max/kg in ultra-marathon runners. (**a**) miR-125a-5p; (**b**) Let-7e; (**c**) miR-126, * Comparison is significant at the 0.05 level.

To analyze the correlation between studied miRNAs expression and VO_2_max/kg in ultra-marathon runners, we divided VO_2_max/kg levels based on quartiles. Ultra-marathon runners with the highest quartile of VO_2_max/kg had 23-fold lower miR-126 expression in comparison to subgroups with lower VO_2_max/kg (*p* = 0.017). No significant differences were found for miR-125a-5p and Let-7e expressions (*p* = 0.059; *p* = 0.262, respectively) (Figure 4).

## 4. Discussion

This study assessed the effects of physical fitness on the levels of miRNAs associated with inflammation, endothelial function, and atherosclerosis in a unique population of high-level long-term ultra-marathon runners. Firstly, our study showed that miR-125a-5p expression was remarkably upregulated in well-trained individuals in comparison to a control group of subjects not engaged in any regular sport activity. In a previous study even a 2 min all-out running HIIT in young healthy moderately trained individuals was sufficient to increase the expression of circulating miR-125a-5p, which has vasculo-protective functions [47,48]. However, we found a negative correlation between VO_2_max, which is widely recognized as the best indicator of cardiovascular fitness and aerobic endurance, with both miR-125a-5p and miR-126. Similarly, to our results, the HUNT study also revealed that increased levels of miR-125a were associated with low VO_2_max in male participants [49]. To the best of our knowledge, miRNAs mentioned above, their association with cardiopulmonary fitness parameters and CIMT have not been extensively studied in individuals who participate in ultra-marathon runs and whose weekly training volume exceeds 70 km of running. 

Previously, it was hypothesized that shear stress linked with exercise may stimulate endothelial cells to secrete miRNAs in vivo. Shear stress, particularly when blood flow is disturbed, plays a crucial role in the pathogenesis of the atherosclerotic plaque. Previous in vitro study showed that endothelial cells secrete miR-125a-5p induced by shear stress, which pointed out the vasculoprotective and anti-atherosclerotic potential of miR-125a [48]. Shear stress is essential in controlling the atheroprotective dysfunction of the vessel wall through multifactorial molecular mechanisms that stimulate atherogenesis [50]. Further analysis is needed to confirm the promising value of miRNAs as biomarkers of shear stress in atherosclerosis. HIIT was found to influence the expression of various miRNAs. MiR-125a-5p expression was increased in correlation with HIIT, which may play a role in this phenomenon [48]. However, miR-126 was similarly over-expressed after high-volume training (HVT; 130 min at 55% peak power output-PPO) and sprint-interval training (SIT; 4 × 30 s all-out), but not high-intensity training (HIT; 4 × 4 min at 95% PPO), which may indicate secretion of endothelial miRNAs into the circulation. A study based on comparison of different exercise protocols speculated that hypoxic conditions are more likely to occur during higher intensities, explaining the largest elevation of circulating miR-126 after SIT [51]. Additionally, it was indicated that endurance exercise causes damage to the endothelial cell layer as confirmed by an increase in circulating miR-126 [22]. It was described that miR-126 is packed into endothelial microparticles under condition of endothelial apoptosis and then transported to target vascular smooth muscle cells in in vitro analysis [51]. This miRNA also diminishes endothelium damage through restoration of autophagic flux by suppressing the PI3K/Akt/mTOR signaling in in vitro model of atherosclerosis [52]. It is important to note that, still there are no specific biomarkers which can identify the best exercise protocol or duration. However, studies showed that an intensity or volume-dependent regulation of angiogenesis, hypoxia and inflammation-related circulating miRNAs, suggesting that the exercise dosage may be a crucial set off.

Further analysis of our results showed that the individuals with the highest quartile of VO_2_max had a lower expression of miR-125a-5p, miR-126 and Let-7e, where only miR-126 was significantly decreased. Moreover, we found a negative correlation between vasculo-protective miRNAs and VO_2_max in ultra-marathon runners, namely miR-125a-5p and miR-126. This can be in contradiction to some previous findings that showed a positive correlation between endurance activity, VO_2_max and the expression of certain miRNAs [11,53]. It is of note that miR-126 levels seem to get easily increased in athletes because of hypoxic stress [51,54]. As hypoxic conditions are more likely to occur during training with higher intensities, it may explain the highest increase of circulating miR-126 after sprint short intensive training [55]. Additionally, various endurance exercises induce the expression of miR-126 and circulating levels of miR-126 have been suggested as a new marker of endothelial injury [56]. It can be speculated that in individuals with higher VO_2_max lower hypoxic stress after the same intensity of training can be expected, hence lower expression of miR-126. In the functional analysis, with the use of an in vitro model, it was demonstrated that upregulated miR-126 acts as an angiogenesis suppressor under hypoxia conditions [57]. Thus, lower miR-126 expression in our cohort may suggest that lower expression of miR-126 in individuals with higher VO_2_max serves as an indicator of adaptive processes of enhanced angiogenesis. Of note, highly fit individuals with high VO_2_max are more likely to develop more severe exercise-induced hypoxemia, due to adaptation to exercise at a greater % of VO_2_max [58]. Additionally, in vitro studies revealed that miR-126 expression significantly decreased after hypoxia treatment in a time-dependent manner. It was shown that after 6 h in hypoxic conditions miR-126 expression is significantly decreased, reaching even a 100-fold reduction in expression level 24 h after hypoxia treatment. On the other hand the VEGF expression, which is closely associated with neovascularization, has been found to increase 24 h after hypoxia treatment [57]. It should be noted that the VEGF pathway is also related to hypoxia and was described to be differently activated between groups with low and high VO_2_max. It may play a role in the formation of new capillaries [49,59]. However, it will need to be confirmed in further clinical and experimental studies whether subjects with lower VO_2_max and elevated miR-126 and miR-125a-5p expression have increased activity in hypoxia- and angiogenesis pathways.

Moreover, we found a significant correlation between VO_2_max and CIMT (r = −0.576, *p* = 0.002). It was previously shown by Kang et al. that high VO_2_max is related to a decrease in CIMT in comparison to low VO_2_max, as there was a weak correlation between VO_2_max and CIMT (r = −0.129, *p* < 0.001) [60]. A stronger correlation in our small cohort could be partly explained by higher VO_2_max values (60.97 ± 4.88 mL/kg/min) in comparison with the subgroup with the highest value from the Kang et al. study (49.05 ± 3.47 mL/kg/min). Interestingly, we also observed a positive association between CIMT and the expression of both miR-125a-5p and miR-126. It was described that miR-126 might serve as a biomarker of atherosclerosis as it exerts anti-apoptotic, anti-inflammatory, regulatory effect on lipid metabolism in endothelial cells. Thus, its over-expression in well trained subjects might be a regulatory mechanism and prevent the atherosclerosis progression and development due to the suppression of inflammation in atherosclerotic plaque [61]. It was hypothesized that the miR-126 upregulation might play an anti-atherosclerotic role, and may reduce leukocyte migration from the bloodstream through the endothelium to the vessel wall in vivo [62,63]. Interestingly, it was previously described that miR-125a can be downregulated in patients with atherosclerosis [33]. Thus, upregulation may play a protective function, as it has been reported that miR-125a-5p is able to diminish the expression of inflammatory markers in monocytes [64]. Therefore, both miR-125a-5p and miR-126 show the ability to diminish ox-LDL uptake in endothelial cells and monocytes, and thus may protect against the development of atherosclerosis [65]. Those findings were further confirmed in in vitro studies using human brain microvessel endothelial cells, as it was shown that upregulation of miR-125a-5p preserved endothelial cells against ox-LDL-induced cell death, senescence, ROS generation, and NO reduction. Furthermore, increased expression of miR-125a-5p induced the proliferation and migration of endothelial cells, while reducing leukocyte adhesion, and preventing the influence of ox-LDL on these processes [66,67]. Diminished levels of miR-125a-5p were also found to be associated with endothelial dysfunction in children and it may target genes relevant in the context of abnormal endothelial function [68]. Therefore, it can be speculated that miR-125 family might be upregulated during the early inflammation process, which accompany increased intima–media thickness, even though it is downregulated during neointima and plaque formation in atherosclerotic plaques of coronary atherosclerosis patients [33,69]. The above-mentioned observation is especially interesting in light of the previously described long-term effect of endurance exercise training on coronary atherosclerosis. In the athletic cohort coronary artery calcification (CAC) may be present in up to 71% of individuals and coronary artery calcification score CACS > 100 in up to 36%. Moreover, CAC is more common in male marathon runners with additional risk factors like history of hypertension, history of smoking than in athletes without cardiovascular risk factors [10]. What is more important for the marathon runners’ population is that the prevalence of CAC is remarkably higher in the most active athletes in comparison to the least active athletes [10,70].

## 5. Limitations 

One of the major limitations of our study is that we measured miRNAs in a relatively small cohort of 30 ultra-marathon runners. However, according to our best knowledge, this study recruited the largest number of individuals out of any study assessing the expression of circulating miRNAs in athletes. Other limitations are related to the demographic characteristic of individuals regarding gender, and thus our results are not applicable to females. Another limitation is that withdrawing blood between 12–24 h after the last training is a long window as it may affect the expression levels of miRNAs. Moreover, we also measured only selected and known miRNAs using a RT-qPCR, and thus potentially other relevant miRNAs could not be analyzed in this study. As long-term follow-up was not available in this study, no firm conclusions about long-term effects can be drawn from the available data. Moreover, we cannot conclusively state whether the observed changes are harmful or whether any study participant will develop clinically significant atherosclerosis in the future.

## 6. Conclusions

The study outcomes suggest a possible role of circulating miRNAs as a biomarker of adaptation in the ultra-marathon runner’s cardiovascular system. A positive correlation between miR-125a-5p and miR-126 and CIMT may suggest the possibility that both miRNAs serve as a biomarker for early pathological changes leading to atherosclerosis burden in athletes, however, their predictive value in this setting should be further evaluated. Furthermore, the association between circulating miRNAs and traditional CVD risk factors suggest a potential of these miRNAs as early biomarkers of future cardiovascular health. Future studies will be essential to investigate the impact of extensive and regular physical exercise on miR-125a-5p and miR-126 uptake or production in the heart and their effect on adaptation of the cardiovascular system to extreme exercise loading.

## Figures and Tables

**Table 1:** Characteristics of ultra-marathon runners.

Parameter	Ultra-Marathon Runners *n* = 30, Median (IQR)
Years of running (running history)	9 (7–15)
Years of ultra-training	6 (5–8)
Age at start of ultra-running	34 (29–39)
Total covered distance (km)	25,000 (20,000–40,000)
Weekly running distance (km)	80 (70–90)
Number of ultra-races completed	15 (10–27.5)
Number of ultra-races during previous year	5.5 (4–9)
Number of completed ultra-races >100 km	3.5 (2–7)
Best place achieved in an ultra-race	5 (1–13)
Longest run (km)	150 (106–246)
Most frequent ultra-race distance (km)	100 (70–100)

Data are reported as median and interquartile range. Abbreviations: standard deviation, SD; interquartile range.

**Table 2:** Participants baseline characteristics and results.

Parameter	Ultra-Marathon Runners (*n* = 30)	Control Group (*n* = 9)	*p* Value
Age (years)	40.93 ± 6.57	40.0 ± 8.32	0.76
Height (cm)	178 ± 5	179 ± 4	0.51
Weight (kg)	71.9 ± 4.7	83 ± 6.1	0.12
BMI (cm/m^2^)	22.07 ± 1.54	26.1 ± 1.5	0.13
Systolic BP (mmHg)	128 ± 6	126 ± 7	0.78
Diastolic BP (mmHg)	78 ± 6	80 ± 5	0.35
Hct (%/100)	0.43 ± 0.03	0.45 ± 0.02	**0.01** *
Resting HR (bpm)	54.9 ± 9.2	69.6 ± 11.0	**0.005** *
VO_2_ max (L/min)	4.43 ± 0.44	3.63 ± 0.63	**0.005** *
VO_2_ max (mL/min/kg)	61.0 ± 4.9	40.2 ± 4.6	**<0.001** *
VE max (L)	150.4 ± 16.9	127.9 ± 25.3	0.03*
RER max	1.08 ± 0.05	1.10 ± 0.04	0.17
LVEDVI (mL/m^2^)	110 ± 15	78 ± 10	**<0.0001** *
LVESVI (mL/m^2^)	39 ± 9	27 ± 4	**<0.0001** *
LVSVI (mL/m^2^)	71 ± 9	51 ± 9	**<0.0001** *
LVEF (%)	65 ± 5	66 ± 5	0.59
LVMI (g/m^2^)	83 ± 11	66 ± 11	**0.001** *
RVEDVI (mL/m^2^)	125 ± 20	88 ± 12	**<0.0001** *
RVESVI (mL/m^2^)	52 ± 11	37 ± 6	**<0.0001** *
RVEF (%)	59 ± 4	58 ± 5	0.61
RVMI (g/m^2^)	24 ± 3.5	22 ± 5	0.24
IVSD (mm)	11.5 ± 2	10.5 ± 1	0.32
PWD (mm)	10 ± 1	9 ± 1	0.17
Left CIMT	0.07 ± 0.02	0.07 ± 0.01	0.99
Right CIMT	0.08 ± 0.03	0.08 ± 0.01	0.68

Abbreviations: BMI—body mass index, BP—blood pressure, CIMT—carotid intima–media thickness, Hct—hematocrit, HR—heart rate, IVSD—interventricular septal diameter, LVEDVI—left ventricular end-diastolic volume index, LVEF—left ventricular ejection fraction, LVESVI—left ventricular end-systolic volume index, LVMI—left ventricular mass index, LVSVI—left ventricular stroke volume index, PWD—left ventricular posterior wall diameter, RER—respiratory exchange ratio, RVEF—right ventricular ejection fraction, RVEDVI—right ventricular end-diastolic volume index, RVESVI—right ventricular end-systolic volume index, RVMI—right ventricular mass index, RVSVI—right ventricular stroke volume index, VE—minute ventilation, VO_2_ max—maximal oxygen consumption. * *p* values marked with bold indicate statistically significant differences between the groups.

## Data Availability

All datasets used in this study are available to obtain upon request of from the corresponding author.
